# Impact of hemoglobin level on the association between lipid metabolism and gestational diabetes mellitus: A retrospective study

**DOI:** 10.1097/MD.0000000000041778

**Published:** 2025-03-07

**Authors:** Xinxin Yang, Guangya Wang, Rui Jiang, Nairui Zhao, Xiuping Yin, Cuiliu Li

**Affiliations:** a Department of Endocrinology, Cangzhou Central Hospital, Cangzhou, China; b Department of Neonatology, Cangzhou Central Hospital, Cangzhou, China.

**Keywords:** dyslipidemia, gestational diabetes mellitus, gestational hypertension, hemoglobin, prepregnancy BMI

## Abstract

To investigate the relationship of hemoglobin (HGB), dyslipidemia, and prepregnancy body mass index with gestational diabetes mellitus (GDM). This study included 1046 pregnant women who underwent antenatal examinations at our hospital between July 30th, 2018, and July 30th, 2019. Participants were divided into 2 groups: those with GDM and those without GDM. Logistic regression analysis was used to explore associations, and receiver operating characteristic curve analysis was performed to evaluate the predictive ability of hematological parameters for GDM. Subgroup analyses were performed to examine the association between HGB levels and GDM risk in different biochemical parameter subgroups. After adjusting for potential confounding factors, higher hemoglobin level in the first trimesters (HGB1) and hemoglobin levels in the second trimesters (HGB2) were associated with GDM risk. Women with low-density lipoprotein (LDL) level exceeding 2.2 mmol/L who had higher HGB1, HGB2 and hemoglobin levels in the third trimesters (HGB3) showed a significantly higher risk of GDM. Higher HGB1, HGB2, and hemoglobin levels in the third trimester were risk factors for GDM, and this association was significant among women with LDL ≥ 2.2 mmol/L. Our findings highlight HGB level as a potential novel marker for screening GDM risk in the first trimester, particularly among women with dyslipidemia.

## 1. Introduction

Gestational diabetes mellitus (GDM) refers to the onset or first recognition of abnormal glucose tolerance during pregnancy.^[1]^ Following the Hyperglycemia and Adverse Pregnancy Outcome study, the International Association of Diabetes and Pregnancy Study Groups established new diagnostic criteria for GDM.^[2,3]^ These criteria are widely endorsed by guidelines in different countries.^[4–8]^ GDM carries significant maternal and fetal risks, including increased risk of preterm delivery, delivery of large for gestational age neonate, polyhydramnios, preeclampsia, neonatal hypoglycemia, hyperbilirubinemia, respiratory distress syndrome, and need for admission to a neonatal intensive care unit.^[9,10]^ Pregnancy is a unique physiological state, characterized by changes in glucose metabolism to meet the energy needs of the fetus. Pregnant women develop pathological and physiological changes such as endothelial dysfunction and oxidative stress damage, leading to insulin resistance and metabolic abnormalities. Many women with early GDM in the first trimester of pregnancy do not have evidence of hyperglycemia at 24–28 weeks gestation. A high proportion (15–70%) of women with GDM can be diagnosed early in pregnancy.^[11]^

Hemoglobin (HGB) contributes to increased iron stores, triggering oxidative stress and the production of reactive oxygen species, which damage pancreatic β cells and reduce insulin synthesis and secretion.^[12]^ Hemodilution is a physiological phenomenon occurring during pregnancy to improve blood flow to the uterus and fetus, and hemodilution starts during the first trimester.^[13]^ Some studies have shown that higher HGB levels during pregnancy are associated with adverse pregnancy outcomes, including GDM.^[14]^ Few studies have assessed the relationship between hemoglobin level in the second trimesters (HGB2) or hemoglobin level in the third trimesters (HGB3) and the risk of GDM. Advanced maternal age, prepregnancy overweight/obesity, and dyslipidemia are known risk factors for GDM.^[15,16]^ However, the relationship of HGB levels, dyslipidemia, and prepregnancy body mass index (pre-BMI) with GDM is not well characterized in contemporary literature.

This study investigated the temporal changes in HGB levels during pregnancy in GDM and non-GDM women and the association of HGB levels and metabolic markers (such as age, dyslipidemia, and pre-BMI) with GDM.

## 2. Materials and methods

### 2.1. Study population

Pregnant women who delivered at the Cangzhou Central Hospital, Hebei, China between July 30th, 2018, and July 30th, 2019 were eligible for enrollment in this study. The exclusion criteria were as follows: nonsingleton pregnancies; incomplete data regarding the 1st trimester; those who were diagnosed with the following diseases before pregnancy: severe cardiac, hepatic, renal disorders; hematological disorders; hypertension; diabetes; thyroid disorders; malignant tumors; or hyperlipidemia; those who had conceived with assisted reproductive technology; loss to follow-up; and use of drugs affecting HGB level before pregnancy (e.g., glucocorticoids, propylthiouracil, labetalol, methimazole, antibiotics, or levothyroxine). The study protocol was approved by the medical ethics committee of the Cangzhou Central Hospital and complied with the principles enshrined in the Declaration of Helsinki.

### 2.2. Methods

Sociodemographic characteristics of all patients were recorded using a standardized format. These data were used for calculating prepregnancy BMI and recording history of adverse pregnancy outcomes. Fasting blood samples were used to measure laboratory indices. HGB levels were tested in the first, second, and third trimesters. The reference range of HGB was 110–150 g/L (Mindray BC-7500 CS, China). Lipid profile was assessed using venous blood samples collected in the 2nd trimester of pregnancy following overnight fasting. Serum levels of triglycerides (TG), total cholesterol (TC), low-density lipoprotein (LDL), and high-density lipoprotein (HDL) levels were determined using an automatic biochemical analyzer (Hitachi 7180, Japan).

### 2.3. Diagnostic criteria

A 75 g 2-hour oral glucose tolerance test (fasting, 1-hour, and 2-hour) was used to diagnose GDM, and the results were interpreted using the International Association of Diabetes and Pregnancy Study Group criteria. Gestational hypertension (systolic blood pressure ≥ 140 mm Hg or diastolic blood pressure ≥ 90 mm Hg) was diagnosed when hypertension appeared after 20 gestational weeks.

### 2.4. Statistical analysis

Normally distributed continuous variables were presented as mean ± standard deviation, while those with skewed distribution were presented as median (P25–P75). Categorical variables were presented as frequency (percentage). For assessing differences between 2 subgroups without intervention, the Kolmogorov–Smirnov test was applied for normally distributed continuous variables, the Mann–Whitney *U*-test was used for nonnormally distributed variables, and the χ^2^ test was used for categorical variables. The correlations among continuous variables were assessed using Pearson correlation analysis.

The restricted cubic spline (RCS) regression model was used to model the potential nonlinear relations of HGB level with GDM, to adjust for potential confounding factors (age, pre-BMI, gravidity, parity, TG, TC, HDL, and LDL). Receiver operating characteristic curve analysis was used to estimate the area under the receiver operating characteristic curve and 95% confidence interval of HGB-predicted GDM events. Univariate and multivariate logistic regression were conducted to explore the association between HGB and GDM after adjusting for confounders (maternal age, pre-BMI, gravidity, parity, and lipid profile). Subgroup analyses were performed to explore whether the association of HGB with GDM differs among populations with different characteristics. The variables were classified as follows: maternal age (<35, ≥35 years), pre-BMI (<24, ≥24 kg/m^2^), TG (<0.9, ≥0.9 mmol/L), TC (<4.2, ≥4.2 mmol/L), HDL (<1.5, ≥1.5 mmol/L), LDL (<2.2, ≥2.2 mmol/L), Two-tailed *P* values < .05 were considered indicative of statistically significant differences for all tests. SPSS 26.0 (IBM, Armonk) and R4.3.3 were used for data analyses.

## 3. Results

### 3.1. Baseline characteristics of the study population

**Figure 1.**
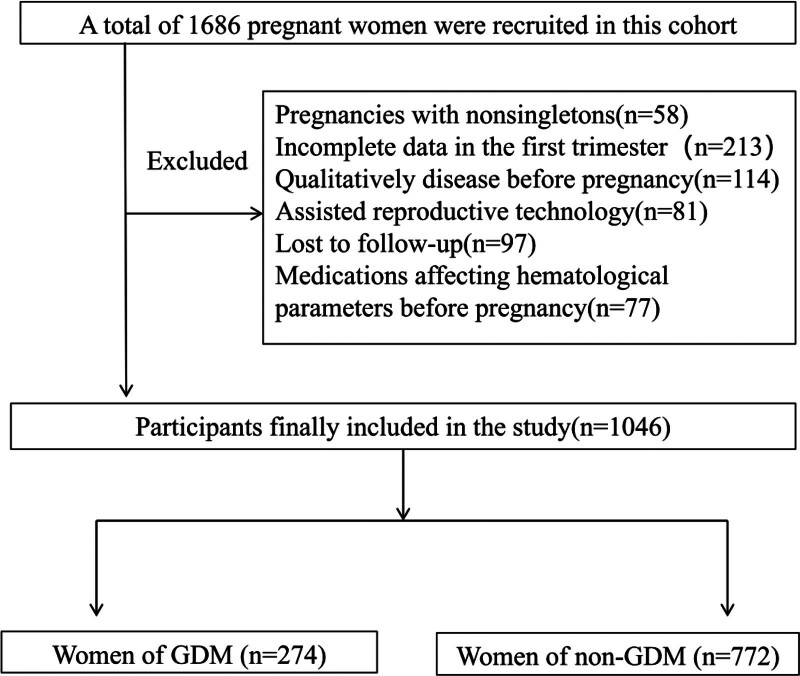
Flowchart of the protocol used to select the study population. A total of 1686 pregnant women who delivered were eligible for enrollment in this study, 1046 participants finally included in the study.

A total of 1686 pregnant women were eligible for this study. Of these, 640 women met the exclusion criteria. Therefore, data pertaining to 1046 women with a singleton pregnancy were included in the analyses (Fig. 1). The clinical characteristics of the study population are summarized in Table 1. At baseline, there were significant differences between pregnant women with and without GDM with respect to age (32.55 ± 4.10 years vs 31.23 ± 3.82 years, *P* < .001), pre-BMI (23.38 ± 3.70 kg/m^2^ vs 21.83 ± 2.96 kg/m^2^, *P* < .001), TG (1.09 [0.80–1.69] mmol/L vs 0.86 [0.67–1.20] mmol/L, *P* < .001), TC (4.48 [3.86–5.24] mmol/L vs 4.06 [3.61–4.62] mmol/L, *P* < .001), LDL (2.43 [2.00–2.93] mmol/L vs 2.09 [1.78–2.50] mmol/L, *P* < .001), and gestational hypertension (7.17% vs 6.09%, *P* = .029).

**Table 1:** Baseline characteristics of all 1046 pregnant women.

Parameters	Total (N = 1046)	GDM (N = 274)	Non-GDM (N = 772)	*P* value
Age (yr), mean ± SD	31.58 ± 3.94	32.55 ± 4.10	31.23 ± 3.82	<.001
Gravidity, median (P25–P75)	2.00 (1.00–2.00)	2.00 (1.00–3.00)	2.00 (1.00–2.00)	.044
Parity, median (P25–P75)	1.00 (1.00–2.00)	1.00 (1.00–2.00)	1.00 (1.00–2.00)	.258
Pre-BMI (kg/m^2^), mean ± SD	22.23 ± 3.24	23.38 ± 3.70	21.83 ± 2.96	<.001
Gestational week, median mean ± SD	39.22 ± 1.27	39.03 ± 1.20	39.29 ± 1.30	.109
Body weight (g), mean ± SD	3300.42 ± 429.42	3337.15 ± 447.56	3287.38 ± 422.32	.076
TG (mmol/L), median (P25–P75)	0.92 (0.69–1.31)	1.09 (0.80–1.69)	0.86 (0.67–1.20)	<.001
TC (mmol/L), Median (P25–P75)	4.15 (3.66–4.80)	4.48 (3.86–5.24)	4.06 (3.61–4.62)	<.001
HDL (mmol/L), median (P25–P75)	1.51 (1.31–1.75)	1.52 (1.30–1.79)	1.51 (1.31–1.74)	.596
LDL (mmol/L), median (P25–P75)	2.18 (1.81–2.64)	2.43 (2.00–2.93)	2.09 (1.78–2.50)	<.001
Gestational hypertension (n, %)	75, 7.17	28, 10.3	47, 6.09	.029

*P* value stands for statistically significant value with *P* < .05.

GDM = gestational diabetes mellitus, HDL = high-density lipoprotein, LDL = low-density lipoprotein, pre-BMI = prepregnancy body mass index, SD = standard deviation, TC = total cholesterol, TG = triglycerides.

### 3.2. Hemoglobin levels with and without GDM

We assessed the dynamic changes in HGB level from the first to third trimesters and found significant differences in HGB levels in pregnant women with and without GDM. Women with GDM had significantly higher HGB 1, HGB2 and HGB3 than women without GDM (134.00 ± 8.04 g/L vs 130.11 ± 8.07 g/L, 119.95 ± 7.98 g/L vs 117.20 ± 8.24 g/L, 125.63 ± 9.96 g/L vs 122.59 ± 9.84 g/L, respectively; *P* < .001 for all) (Table 2).

**Table 2:** Hemoglobin levels with and without GDM in the first, second, and third trimesters.

	Total (N = 1046)	GDM (N = 274)	Non-GDM (N = 772)	*P* value
HGB1 (g/L), mean ± SD	131.13 ± 8.24	134.00 ± 8.04	130.11 ± 8.07	<.001
HGB2 (g/L), mean ± SD	117.92 ± 8.26	119.95 ± 7.98	117.20 ± 8.24	<.001
HGB3 (g/L), mean ± SD	123.39 ± 9.96	125.63 ± 9.96	122.59 ± 9.84	<.001

GDM = gestational diabetes mellitus, HGB1 = hemoglobin level in the first trimesters, HGB2 = hemoglobin level in the second trimesters, HGB3 = hemoglobin level in the third trimesters, SD = standard deviation.

### 3.3. Relationship between hemoglobin levels and GDM

**Figure 2.**
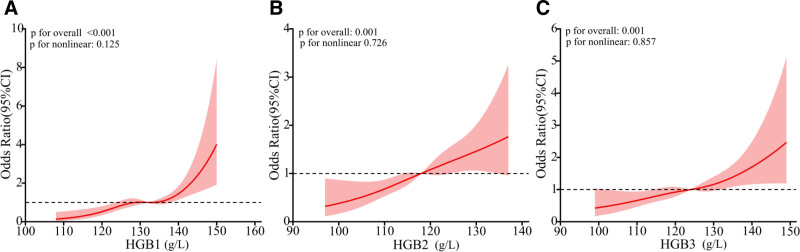
The restricted cubic spline regression model was implemented to investigate the exposure-response association between HGB level and the prevalence of GDM. (A) The association of HGB in the first trimesters with the risk of GDM. (B) The association of HGB in the second trimesters with the risk of GDM. (C) The association of HGB in the third timesters with the risk of GDM. All of models were to adjusted for potential confounding factors (age, pre-BMI, gravidity, parity, TG, TC, HDL, and LDL). The lines indicate estimated ORs, and the light red areas represent 95% CI. The increase in HGB level was associated with a gradual increase in the risk of GDM. *P* value < .05 indicates statistical significance. GDM = gestational diabetes mellitus, HDL = high-density lipoprotein, HGB = hemoglobin, HGB1 = hemoglobin in the first trimesters, HGB2 = hemoglobin in the second trimesters, HGB3 = hemoglobin in the third timesters, LDL = low-density lipoprotein, OR = odds ratio, pre-BMI = prepregnancy body mass index, TC = total cholesterol, TG = triglyceride.

A random-effects RCS model with 3 knots was used to test for potential nonlinearity in the association of HGB levels with GDM incidence after adjusting for age, pre-BMI, gravidity, TG, TC, HDL, and LDL. Based on the RCS curve, HGB was associated with the occurrence of GDM (*P* for overall trend < .001). The increase in HGB level was associated with a gradual increase in the risk of GDM (Fig. 2). Before the median level, the increase in HGB level was associated with a relatively rapid increase in GDM risk, but there was no nonlinear association (*P* for nonlinear = .125, *P* for nonlinear = .726, and *P* for nonlinear = .857).

**Figure 3.**
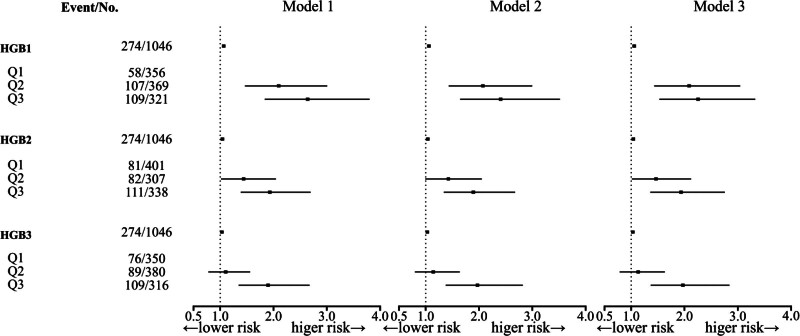
Univariate and multivariate logistic regression were further used to explore the relationship between HGB level at different time points (grouped according to tertiles of HGB level) and the risk of GDM by building multiple models. Model 1 did not account for any confounding factor; model 2 was adjusted for age, pre-BMI, gravidity, and parity; and model 3 was adjusted for TG, TC, HDL, and LDL based on model 2. Higher HGB level was associated with an increased risk of GDM. GDM = gestational diabetes mellitus, HDL = high-density lipoprotein, HGB = hemoglobin, HGB1 = hemoglobin in the first trimesters, HGB2 = hemoglobin in the second trimesters, HGB3 = hemoglobin in the third timesters, LDL = low-density lipoprotein, OR = odds ratio, pre-BMI = prepregnancy body mass index, TC = total cholesterol, TG = triglyceride.

Univariate and multivariate logistic regression were further used to explore the relationship between HGB level at different time points (grouped according to tertiles of HGB level) and the risk of GDM by building multiple models, the results are presented in Figure 3. Model 1 did not account for any confounding factor; model 2 was adjusted for age, pre-BMI, gravidity, and parity; and model 3 was adjusted for TG, TC, HDL, and LDL based on model 2. In model 3, higher hemoglobin level in the first trimesters (HGB1: 97–128 g/L, 129–135 g/L, and > 135g/L) were associated with an increased risk of GDM (odds ratio [OR] = 1.06, *P* < .001; OR = 2.09, *P* < .001; OR = 2.26, *P* < .001, respectively). Higher HGB2 (HGB2: 88–115 g/L, 116–143 g/L, and > 143 g/L) were also associated with an increased risk of GDM (OR = 1.04, *P* < .001; OR = 1.47, *P* = .043; OR = 1.94, *P* < .001, respectively) in model 3.

**Figure 4.**
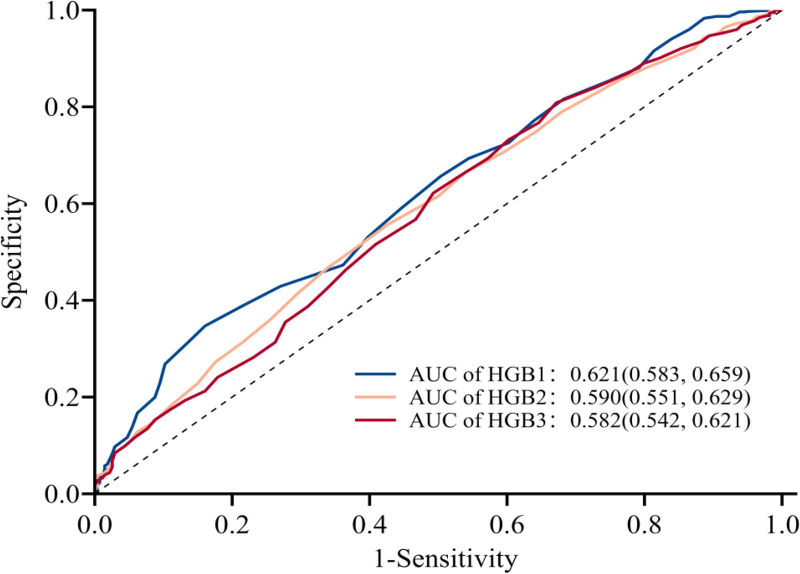
ROC curve analysis for HGB level in predicting GDM. The blue lines represent HGB1, the pink lines represent HGB2, the purple lines represent HGB3. The AUCs of HGB1, HGB2, and HGB3 all were > .5. AUC = area under the ROC curve, GDM = gestational diabetes mellitus, HDL = high-density lipoprotein cholesterol, HGB = hemoglobin, HGB1 = hemoglobin in the first trimesters, HGB2 = hemoglobin in the second trimesters, HGB3 = hemoglobin in the third timesters, ROC = receiver operating characteristics.

Table 3 and Figure 4 show the predictive values of HGB level for GDM in late pregnancy. The area under the receiver operating characteristic curves of HGB1, HGB2, and HGB3 were 0.621, 0.590, and 0.582, respectively (*P* < .001), and the Jordan index was 0.186, 0.130, and 0.136, respectively.

**Table 3:** Value of various inflammatory factors in predicting gestational diabetes mellitus in the third trimester of pregnancy.

Category	AUC (95% CI)	*P* value	Cutoff value	Sensitivity (%)	Specificity (%)	Jordan index
HGB1	0.621 (0.583–0.659)	<.001	127.500	0.839 (0.796–0.883)	0.347 (0.314–0.381)	0.186
HGB2	0.590 (0.551,0.629)	<.001	118.500	0.573 (0.514–0.632)	0.557 (0.522–0.592)	0.186
HGB3	0.582 (0.542,0.621)	<.001	130.500	0.328 (0.273–0.384)	0.808 (0.781–0.836)	0.136

AUC = area under the ROC curve, HGB1 = hemoglobin level in the first trimesters, HGB2 = hemoglobin level in the second trimesters, HGB3 = hemoglobin level in the third trimesters.

### 3.4. Association of HGB levels with GDM risk in different subgroups based on biochemical parameters

**Figure 5.**
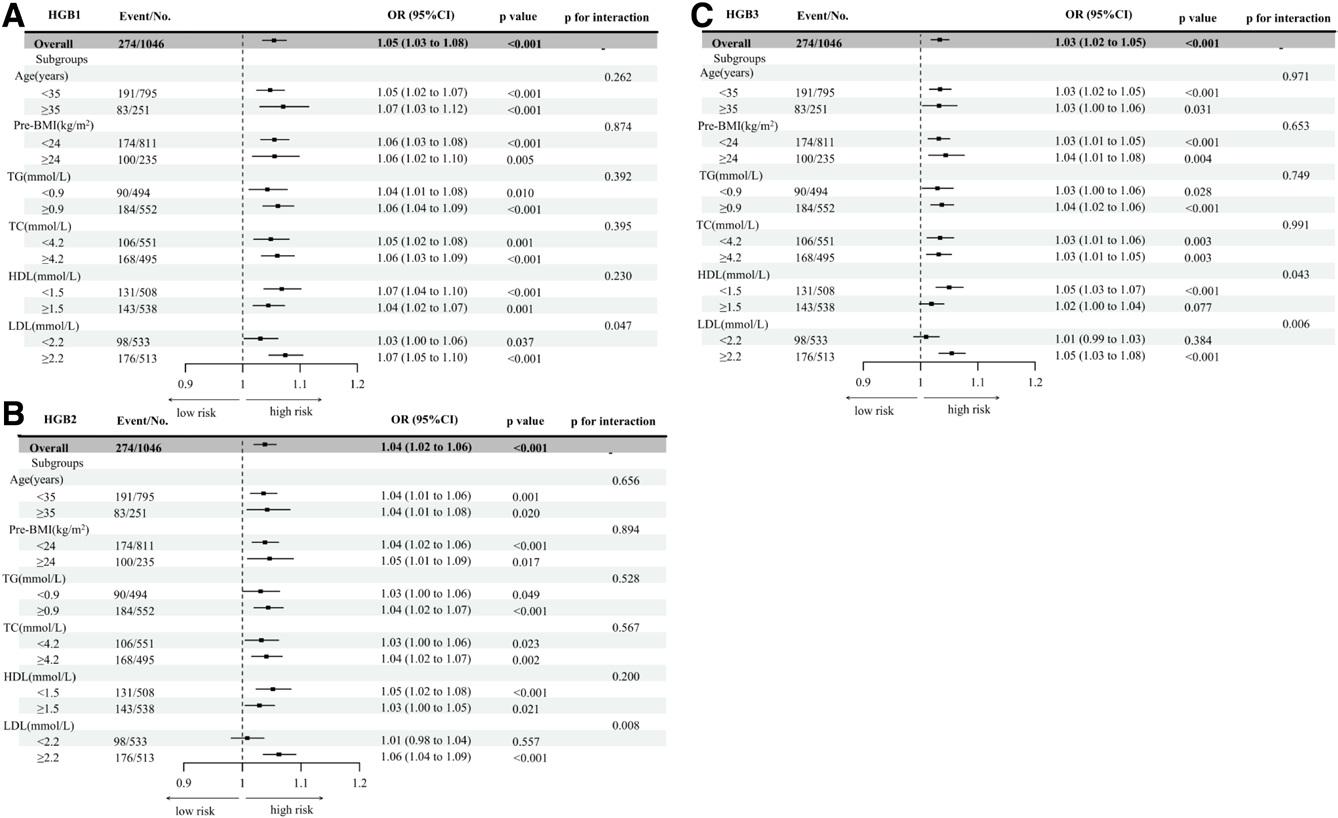
Forest plots were drawn to visualize the correction HGB level with GDM risk in different biochemical parameters subgroup. (A) Forest plots representing the effect sizes and their 95% CI correction HGB level in the first trimesters with GDM risk. (B) Forest plots representing the effect sizes and their 95% CI HGB level in the second trimesters with GDM risk. (C) Forest plots representing the effect sizes and their 95% CI correction HGB level in the third trimesters with GDM risk. Adjusted for age, pre-BMI, gravidity, parity TG, TC, HDL, and LDL. *P* for interaction < .05 indicates statistical significance. GDM = gestational diabetes mellitus, HDL = high-density lipoprotein, HGB = hemoglobin, HGB1 = hemoglobin in the first trimesters, HGB2 = hemoglobin in the second trimesters, HGB3 = hemoglobin in the third timesters, LDL = low-density lipoprotein, pre-BMI = prepregnancy body mass index, TC = total cholesterol, TG = triglyceride.

A subgroup analysis was conducted in which the adjusted variables were the same as in model 3 (adjusted for age, pre-BMI, gravidity, parity, TG, TC, HDL, and LDL). A forest plot of the results is presented in Figure 5. Age was stratified into < 35 and ≥ 35 years according to the standard for advanced maternal age on pregnancy outcomes, and pre-BMI was stratified into < 24 kg/m^2^ and ≥ 24 kg/m^2^ based on the Chinese standard.^[17,18]^ Pregnancy being a hyperlipidemic state, there is no agreed threshold for pathological hyperlipidemia in pregnancy^[19]^; therefore the lipid parameters were stratified by median values. The interaction between HGB1, HGB2, and HGB3 and LDL for GDM risk was significant (*P* for interaction = .047, *P* for interaction = .008, *P* for interaction = .006, respectively). In addition, the interaction between HGB3 and HDL for GDM risk was significant (*P* for interaction = .043). There were no effect modifications by other metabolic markers such as age, pre-BMI, TG, TC, and HDL (*P* for interaction > .05).

## 4. Discussion

In the present study, we assessed the dynamic changes in HGB levels from early to late pregnancy and found significant differences in HGB levels between GDM and non-GDM pregnant women. The dynamic changes in HGB levels have been attributed to inflammatory response, elevated plasma volume, and hemodilution, leading to lower HGB, consistent with our findings.^[20]^ This finding is of clinical relevance, as it may help identify women at risk of GDM, allowing for early interventions to decrease the risk of GDM in future pregnancies. Chronic low-grade inflammation is linked with most of the well-established risk factors for GDM.^[21]^ High HGB levels can increase leukocyte-endothelial adhesion,^[22]^ increasing blood viscosity, a risk factor for insulin resistance, suggesting a potential role in glucose metabolism regulation and a possible value as a diagnostic biomarker.^[23]^

In the present study, higher HGB1 and HGB2 were associated with GDM even after adjusting for potential confounders. In addition, elevated HGB1 and HGB2, not HGB3, were significant predictors of GDM. Consistent with our findings, Damiri et al^[24]^ found that the Hb level is a strong predictor of pregnancy complications in Palestinian pregnant women residing in the north of the West Bank. Another study conducted in Pakistan suggested that the Hb level at registration could be used to predict the risk of GDM and hypertension among women with no previous history of these conditions.^[25]^ Previous studies have evaluated the relationship between GDM and HGB, but the results have been inconsistent. In a study of 1027 Chinese pregnant women, elevated HGB1 and HGB2 were associated with a higher risk of GDM.^[26]^ A case-control study found that higher HGB1 and HGB2 are an independent risk factor for GDM.^[27]^ However, some studies have found a mild negative correlation between HGB and glycated hemoglobin levels.^[28]^ In contrast, another study has found an association between GDM and maternal anemia.^[29]^ A Korean study identified an association between high prepregnancy hemoglobin levels and GDM risk.^[30]^ A study of 600 pregnant women in Iran found no significant difference in RBC counts between GDM and non-GDM groups^[31]^ but in another Iranian study, the mean hemoglobin level in women with GDM was significantly higher than in healthy women.^[32]^ The Avon Longitudinal Study of Parents and Children found that higher maternal HGB is a potential risk factor for adverse pregnancy outcomes including GDM.^[33]^

Our findings are consistent with most studies conducted, showing an association between higher HGB levels and GDM. In our study, HGB was associated with the occurrence of GDM (*P* for overall trend < .001). The RCS curve showed no nonlinear association between HGB and GDM risk. In addition, we did not find a protective effect of lower HGB levels against the risk of GDM in the Chinese population, which was contrary to the findings reported by Niina Sissala and Auvinen.^[26,34]^

Compared with previous studies, an innovative aspect of the present study was the exploration of the impact of HGB level on the correlation between metabolic parameters and GDM during early, middle, and late pregnancy, and the interaction analysis. Women with higher HGB1 and HGB2 showed a higher risk of GDM and this association was significant among women with LDL levels exceeding 2.2 mmol/L. Women with higher HGB3 were at higher risk of GDM and this association was significant among women with LDL levels exceeding 2.2 mmol/L and HDL levels below 1.5 mmol/L. The analyses found a statistically significant increase in the rates of diabetes mellitus after the age of 35 years^[35]^ Yong et al^[36]^ found that higher HGB levels in the first trimester were associated with a higher risk of GDM among women aged ≥ 35 years and those who were overweight or obese. However, there was no effect modification by age, pre-BMI, and other parameters in the present study. The correlation between inflammation biomarkers and oxidative stress in GDM may vary in different populations due to the differences in dietary patterns and other factors such as maternal age, body weight, smoking, and lifestyle factors. Heme-mediated oxidative modification of LDL can lead to endothelial cytotoxicity, and at sublethal doses, the expression of stress-response genes, oxidative stress, and inflammation contribute to the development of GDM.

Some limitations of our study should be considered. This was a single-center study with a relatively small sample size. Further research is required to explore the potential mechanisms linking HGB level with GDM risk and identify interventions targeting HGB to reduce the risk.

## 5. Conclusions

In this study, HGB levels showed dynamic changes during first, second, and third trimesters of pregnancy. Higher HGB levels in the first, and second trimesters were associated with a higher risk of GDM. Women with elevated HGB levels in the first, second, and third trimesters were at higher risk of GDM, and this association was significant among women with LDL levels exceeding 2.2 mmol/L. Our findings highlight HGB levels as a novel marker for screening GDM risk in the first trimester. Higher iron status in pregnancy may be a risk factor for GDM among women with dyslipidemia.

## Author contributions

**Formal analysis:** Xinxin Yang.

**Methodology:** Xinxin Yang, Guangya Wang.

**Software:** Xinxin Yang.

**Writing – original draft:** Xinxin Yang.

**Data curation:** Rui Jiang, Nairui Zhao, Xiuping Yin.

**Conceptualization:** Cuiliu Li.
